# Staged management of infected diabetic foot ulcers: a 300-patient cohort study on prognostic grading, pathogen dynamics, and individualized risk prediction

**DOI:** 10.3389/fmed.2026.1809285

**Published:** 2026-03-27

**Authors:** Yuce Zhang, Jun Zhang

**Affiliations:** The Fourth Hospital of Harbin Medical University, Harbin, China

**Keywords:** amputation, diabetic foot infection, diabetic foot ulcer, IWGDF/IDSA classification, nomogram, osteomyelitis, risk prediction, survival analysis

## Abstract

**Objective:**

This study aimed to evaluate the IWGDF/IDSA classification for prognostic stratification in diabetic foot infections (DFIs) and to develop and validate a clinical prediction model for individualized risk assessment.

**Methods:**

In this retrospective study of 300 patients with DFIs (2020–2024), infection severity was graded as Grade 2 (mild), 3 (moderate), 3(O) (moderate with osteomyelitis), or 4 (severe). We analyzed baseline characteristics, microbiological profiles, treatment responses, and performed time-to-event analyses for ulcer healing and limb salvage. A nomogram predicting 6-month non-healing risk was developed using Cox regression, with performance assessed by C-index and calibration. Subgroup and sensitivity analyses (including competing-risk models) were conducted to test robustness.

**Results:**

Higher infection grades correlated with worse glycemic control (HbA1c: 9.8% in Grade 4 vs. 8.1% in Grade 2), higher peripheral arterial disease (PAD) prevalence (64.3% vs. 22.4%), and a pathogen shift from Gram-positive cocci (87.2% in Grade 2) to Gram-negative bacilli (*Pseudomonas aeruginosa* 52.2%) and polymicrobial infections (60.9%) in Grade 4 (all *p* < 0.001). Kaplan–Meier analysis revealed a severity-dependent gradient in outcomes: median healing time prolonged from 6.0 weeks (Grade 2) to 18.0 weeks (Grade 4), while the 24-week cumulative amputation rate escalated to 66.7% in Grade 4. Osteomyelitis (Grade 3(O)) constituted a distinct high-risk subgroup, with outcomes approximating Grade 4. A nomogram incorporating infection grade, PAD, HbA1c ≥ 9%, and revascularization status demonstrated good discrimination (C-index 0.82, bootstrap-corrected 0.80) and calibration (Hosmer–Lemeshow *p* = 0.342), maintained in temporal validation (C-index 0.79). Subgroup analysis confirmed a significant interaction between infection grade and revascularization, with the greatest benefit in severe PAD patients (interaction *p* = 0.03). Sensitivity analyses, including competing-risk models, affirmed the robustness of the severity–outcome association.

**Conclusion:**

The IWGDF/IDSA classification effectively stratifies DFI patients by integrated metabolic, vascular, microbiological, and prognostic risk. Osteomyelitis warrants distinct management. The developed nomogram provides a validated tool for individualized risk prediction, facilitating early targeted intervention. These findings advocate for a severity-guided, multidisciplinary treatment paradigm to improve outcomes.

## 1 Introduction

Diabetic foot ulcers (DFUs) are among the most serious chronic complications of diabetes, with a lifetime incidence of up to 34% (1) and an increasingly younger affected population. DFUs arise from the interplay of peripheral neuropathy, peripheral arterial disease (PAD), and impaired immune function, typically presenting as skin breakdown, infection, or tissue necrosis that may extend to bone or joints. These lesions markedly increase the risk of amputation—with reported rates up to 33%—and lead to disability in approximately 20% of patients (2, 3), representing a major public health challenge.

Once a DFU becomes infected, the resulting diabetic foot infection (DFI) usually begins with microbial colonization following disruption of the skin barrier and progresses under conditions of hyperglycemia, microvascular dysfunction, and impaired neutrophil activity (4). Notably, the clinical manifestations of DFI may be masked by neuropathy, PAD, or immunosuppression; some patients develop deep tissue involvement or osteomyelitis without typical signs of inflammation (5, 6). Thus, infection assessment should not rely solely on culture results but must integrate wound characteristics, systemic symptoms, and imaging evidence (7).

The severity of DFI is closely related to its anatomical spread. The foot’s fascial compartments facilitate proximal extension of infection along tendons and fascial planes. When inflammatory pressure increases within these closed spaces, ischemic necrosis may ensue, accelerating disease progression (8, 9). Patients with chronic kidney disease, poorly controlled diabetes, or recurrent ulcers are at even higher risk for necrotizing or systemic infections (10). Although many infections begin superficially, without timely intervention, they may rapidly invade deeper soft tissues, joints, or bone, posing a serious threat to limb and life (11).

Current international guidelines recommend staging DFIs based on infection extent, depth, and systemic involvement, and tailoring treatment accordingly—including antimicrobial therapy, debridement, revascularization, and multidisciplinary management (12). However, large-scale, stratified clinical evidence on stage-based treatment of infected DFUs remains limited in China. To address this gap, we retrospectively analyzed 300 patients with DFU-associated infections using the IWGDF/IDSA classification system (13, 14) to stratify infection severity, guide individualized treatment, and evaluate clinical outcomes. This study aims to provide evidence for standardized, stepwise management of DFIs, optimize treatment pathways, reduce amputation rates, and improve patient prognosis.

## 2 Materials and methods

### 2.1 Study design

This was a single-center, retrospective observational cohort study. The study aimed to systematically evaluate the real-world application, microbiological characteristics, treatment response, and long-term clinical outcomes of a stage-based treatment strategy guided by the International Working Group on the Diabetic Foot/Infectious Diseases Society of America (IWGDF/IDSA) clinical classification system in patients with diabetic foot infection (DFI). The study protocol was designed and conducted in strict accordance with the principles of the Declaration of Helsinki and received formal approval from the hospital’s Institutional Review Board prior to initiation (Approval No. 2019–701). As this study involved secondary analysis of existing, anonymized clinical data without new interventions, and all data collection and processing adhered to strict confidentiality agreements, the ethics committee granted a waiver for obtaining individual patient informed consent. However, all patients admitted to the hospital had signed a general consent form permitting the use of their de-identified medical data for retrospective medical research.

### 2.2 Study subjects and screening process

#### 2.2.1 Study population and source

The study population consisted of consecutive patients who presented to the hospital’s Department of Endocrinology and Metabolism, Vascular Surgery, and Wound Care Center between 1 January 2020 and 31 December 2024 for diabetic foot ulcers (DFU) complicated by infection. Potential subjects were initially identified through the hospital’s electronic medical record system using International Classification of Diseases, Tenth Revision (ICD-10) codes for diabetic foot and related complications, supplemented by cross-referencing departmental inpatient and outpatient registries to ensure comprehensive inclusion.

#### 2.2.2 Sample size estimation

To ensure adequate statistical power, a formal sample size calculation was performed during the study design phase. Based on preliminary literature review and pilot data from our center, we assumed a 6-month ulcer non-healing rate of approximately 40% in patients with moderate infection (Grade 3). To detect a clinically meaningful absolute difference of at least 15% in healing rates between different infection grades (e.g., Grade 2 vs. Grade 4) with a two-sided significance level (*α*) of 0.05 and a power (1-β) of 80%, the formula for comparing two proportions was used. The calculation indicated a requirement of at least 70–90 patients per major comparison group. Considering the planned division into four subgroups and subsequent multivariate analyses, to avoid underpowered analyses, the total sample size target was set at 300 patients. This number was deemed sufficient not only for the primary analyses but also for the planned subgroup analyses and predictive model development.

#### 2.2.3 Inclusion and exclusion criteria

Explicit, operational inclusion and exclusion criteria were established. All initially screened medical records were independently reviewed by two researchers.

*Inclusion Criteria:*

1. Age ≥18 years.
2. A confirmed diagnosis of type 2 diabetes mellitus, with a documented disease duration of more than 6 months.
3. Presence of an active, full-thickness skin ulcer distal to the ankle, persisting for at least 2 weeks.
4. Clinical diagnosis of diabetic foot infection, defined as the target ulcer exhibiting at least two of the following local signs or symptoms of inflammation (erythema extending beyond the ulcer margin, swelling, tenderness or spontaneous pain, purulent discharge, or markedly increased local warmth) in the absence of alternative non-infectious explanations (e.g., acute exacerbation of a purely neuropathic ulcer, Charcot neuro-osteoarthropathy, and tophus ulceration).
5. Availability of complete electronic medical records from admission through follow-up, with no missing key data (demographics, laboratory tests, imaging reports, treatment regimens, and post-discharge follow-up records), and a minimum effective post-operative or post-discharge follow-up period of 4 weeks.

*Exclusion Criteria* were designed to exclude conditions that might confound infection assessment, treatment response, or outcome determination:

1. Diagnosis of type 1 diabetes, gestational diabetes, or other specific types of diabetes.
2. Foot lesions definitively attributable to non-infectious etiologies, such as uninfected pure neuropathic ulcers, acute Charcot neuro-osteoarthropathy, skin breakdown due to acute gout, and psoriatic lesions.
3. Primary or metastatic malignancy at the ulcer site, or radiation-induced ulcers.
4. Pregnancy or lactation.
5. Severe psychiatric illness, dementia, or other cognitive impairment precluding reliable clinical assessment or history provision.
6. Missing key information in the medical records required to determine the IWGDF/IDSA infection grade, unclear initial antimicrobial regimen, or undetermined primary outcome status (healing/amputation).

#### 2.2.4 Screening flowchart and final cohort

**Figure 1:**
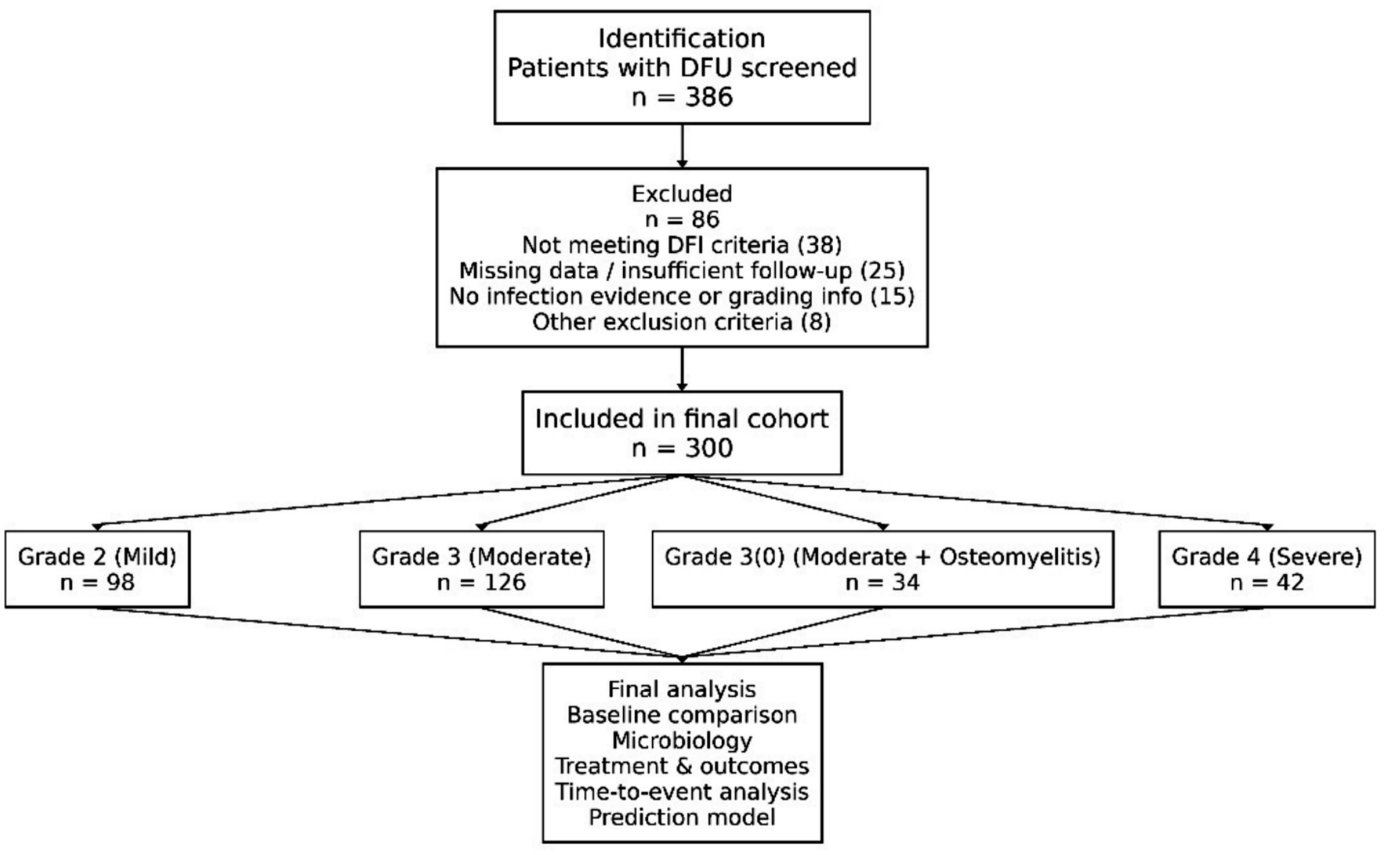
Study enrollment flowchart.

The screening process strictly adhered to the pre-specified criteria and was clearly illustrated using a flowchart (Figure 1). Of the 386 initially identified potentially eligible patients, 300 were ultimately included in the final analysis cohort after sequential screening. Major reasons for exclusion and corresponding numbers were as follows: 38 patients did not meet the clinical diagnostic criteria for DFI (e.g., simple ulcer without clear signs of infection); 25 patients had critical missing baseline data (e.g., HbA1c and vascular examination results) or follow-up information; 15 patients had incomplete or contradictory clinical/imaging evidence required for infection severity grading; and 8 patients met other exclusion criteria (e.g., concomitant lower limb tumor [*n* = 3], pregnancy [*n* = 2], and severe cognitive impairment [*n* = 3]). The final cohort of 300 patients constituted the study population and was allocated into four study subgroups based on their admission infection severity.

### 2.3 Data collection and variable definitions

#### 2.3.1 Data sources and collection process

All data were retrospectively extracted from the hospital’s comprehensive electronic information systems, including the electronic medical record system, laboratory information system, picture archiving and communication system, and surgical anesthesia system. To ensure accuracy and consistency, a standardized data collection form was designed. Data extraction was performed independently by two trained research coordinators not involved in the clinical management of the patients. Discrepancies or contradictions in the records were resolved by consulting original documentation, consulting the attending physician, or arbitration by a third senior researcher.

#### 2.3.2 Baseline characteristics and clinical variables

Collected baseline data included: (1) *Demographics*: age, sex. (2) *Diabetes-related metrics*: duration of diabetes, most recent glycated hemoglobin (HbA1c) value, current glucose-lowering regimen. (3) *Comorbidities*: Presence of hypertension, chronic kidney disease (defined as an estimated glomerular filtration rate <60 mL/min/1.73 m^2^ according to KDIGO guidelines), peripheral arterial disease (diagnosed based on history, physical examination, and/or ABI/TBI ≤ 0.9), and peripheral neuropathy (based on symptoms, signs, and/or neurophysiological testing) were confirmed using diagnostic codes and medication records. (4) *Ulcer characteristics*: location, size, duration, Wagner grade (if applicable). (5) *Infection-related indicators*: Body temperature, heart rate, respiratory rate, white blood cell count, C-reactive protein, and procalcitonin levels at admission.

#### 2.3.3 Microbiological evaluation

All microbiological specimens were obtained via sharp surgical debridement from deep tissue or bone, with strict avoidance of superficial swabs. Whenever clinically feasible, specimens were collected prior to the initiation of antimicrobial therapy. For patients who had already received antibiotics prior to admission or in whom discontinuation of therapy was not clinically safe (particularly those with severe infections presenting with systemic inflammatory response syndrome), specimens were obtained at the time of the first surgical debridement after admission, and the timing of specimen collection relative to antibiotic administration (pre-treatment, during treatment, or post-treatment) was recorded for subsequent sensitivity analysis. Specimens were promptly delivered to the clinical microbiology laboratory for standard aerobic and anaerobic culture, species identification, and antimicrobial susceptibility testing (using disk diffusion or broth microdilution methods). All isolated pathogens and their susceptibility profiles were recorded. Polymicrobial infection was defined as the isolation of two or more pathogenic microorganisms from the same specimen.

Polymicrobial infection was defined as the isolation of two or more distinct pathogenic microorganisms from the same deep tissue specimen obtained under aseptic conditions during surgical debridement. To ensure that isolates represented true pathogens rather than colonizers or contaminants, the following criteria were applied: (1) Specimen quality requirements: All specimens were obtained via sharp surgical debridement from deep viable tissue (not superficial swabs), after thorough wound cleansing and removal of all exudate and necrotic tissue. Specimens were collected prior to antimicrobial therapy whenever clinically feasible, or the timing of antibiotic exposure was recorded. (2) Quantitative thresholds: Isolates were considered clinically significant if they demonstrated growth above laboratory-defined thresholds (typically ≥10^4^ CFU/g for tissue specimens) and were morphologically consistent on culture media. (3) Clinical correlation: Isolates were classified as contaminants if they represented typical skin commensals (e.g., coagulase-negative staphylococci, Corynebacterium species, *Cutibacterium acnes*, and Micrococcus species) in the absence of clinical signs of invasive infection or if they were present only in small numbers alongside dominant pathogens. Conversely, organisms such as *Staphylococcus aureus*, Streptococcus species, Enterococcus species, Enterobacteriaceae, *Pseudomonas aeruginosa*, and obligate anaerobes were consistently regarded as potential pathogens when isolated from appropriate specimens. (4) Repeat isolation: When available, consistent isolation of the same organism from multiple specimens or correlation with Gram stain findings (e.g., presence of neutrophils with intracellular bacteria) supported its designation as a true pathogen.

All culture results were interpreted in conjunction with the patient’s clinical presentation, inflammatory markers, and response to therapy. Cases with ambiguous microbiological findings were reviewed by a multidisciplinary team including infectious disease specialists and clinical microbiologists to reach consensus on pathogen significance.

All *Staphylococcus aureus* isolates were tested for methicillin resistance using both phenotypic and genotypic methods. Phenotypic detection was performed by the cefoxitin disk diffusion method (30 μg) on Mueller–Hinton agar, with results interpreted according to Clinical and Laboratory Standards Institute (CLSI) guidelines (zone diameter ≤21 mm indicating MRSA). For genotypic confirmation, multiplex polymerase chain reaction (PCR) was employed to detect the presence of the mecA gene, which encodes the altered penicillin-binding protein (PBP2a) responsible for methicillin resistance. DNA extraction was performed using a boiling lysis method, and PCR amplification targeted the mecA gene (primers: forward 5′-AAAACTAGGTGTTGGTGAAGATATACC-3′, reverse 5′-GAAAGGATCTGTACTGGGTTAATCAG-3′; product size 147 bp). Isolates were classified as MRSA if they demonstrated resistance by cefoxitin disk diffusion and/or tested positive for the mecA gene. Discrepancies between phenotypic and genotypic results were resolved by repeat testing and, if necessary, PBP2a latex agglutination assay.

Deep tissue and bone specimens were obtained during sharp surgical debridement, placed immediately into sterile containers with sterile saline, and transported to the microbiology laboratory within 2 h for processing. Routine anaerobic culture protocols and dedicated anaerobic transport systems (e.g., Robertson’s cooked meat medium) were not employed. While this method supports recovery of aerobic and facultative anaerobic organisms, it is suboptimal for strict anaerobes and likely contributed to their underdetection in our cohort, particularly in deep infections and osteomyelitis, where anaerobes are commonly implicated. All aerobic and facultative anaerobic isolates were identified using standard microbiological techniques. Gram-positive cocci were identified by colony morphology, Gram stain, catalase test, coagulase test (for *Staphylococcus aureus*), and optochin susceptibility or bile solubility (for *Streptococcus pneumoniae*). Gram-negative bacilli were identified using conventional biochemical panels (e.g., oxidase test, indole test, urease test, citrate utilization, and triple sugar iron agar reactions) and, for a subset of isolates, the VITEK 2 automated system (bioMérieux, France) or API identification strips (bioMérieux). Species-level identification was performed for all clinically significant isolates. For anaerobic organisms, as detailed above, dedicated anaerobic cultures were not routinely performed; therefore, no anaerobic isolates were available for identification. This represents a limitation of the study and likely contributed to the underdetection of obligate anaerobes in our cohort.

Antimicrobial susceptibility testing was performed on all bacterial isolates using the Kirby-Bauer disk diffusion method on Mueller–Hinton agar, following Clinical and Laboratory Standards Institute (CLSI) guidelines. The following antibiotics were tested based on isolate type: for Gram-positive cocci—penicillin, cefoxitin (for MRSA screening), gentamicin, ciprofloxacin, trimethoprim/sulfamethoxazole, clindamycin, erythromycin, rifampicin, linezolid, vancomycin, and teicoplanin; for Gram-negative bacilli—ampicillin, amoxicillin/clavulanate, piperacillin/tazobactam, cefazolin, ceftriaxone, ceftazidime, cefepime, ertapenem, imipenem, meropenem, gentamicin, amikacin, ciprofloxacin, and trimethoprim/sulfamethoxazole. For *Pseudomonas aeruginosa*, additional anti-pseudomonal agents (tobramycin) were tested. Minimum inhibitory concentrations (MICs) were determined by broth microdilution for selected isolates with equivocal disk diffusion results. Multidrug resistance (MDR) was defined as acquired non-susceptibility to at least one agent in three or more antimicrobial categories. Extended-spectrum *β*-lactamase (ESBL) production in Enterobacteriaceae was screened using the combination disk method (ceftazidime vs. ceftazidime/clavulanate and cefotaxime vs. cefotaxime/clavulanate). Methicillin-resistant *Staphylococcus aureus* (MRSA) was identified by cefoxitin disk diffusion (zone diameter ≤21 mm). All susceptibility testing was performed and interpreted according to the Clinical and Laboratory Standards Institute (CLSI) guidelines (M100 series, current edition during the study period). As dedicated anaerobic cultures were not routinely performed, no anaerobic isolates underwent susceptibility testing. All susceptibility data are presented in Supplementary Table 1.

#### 2.3.4 Documentation of treatment measures

Detailed records were made of all key therapeutic interventions received by each patient during the study period: (1) *Surgical interventions*: Number, extent, and nature (sharp/mechanical) of debridement procedures; whether bone resection or amputation was performed (specifying the level). (2) *Revascularization*: Whether the patient underwent lower extremity arterial intervention (e.g., angioplasty and stenting) or bypass surgery, and the timing. (3) *Antimicrobial therapy*: Drug class, dosage, and route of administration for initial empirical therapy; subsequent adjustments based on susceptibility results; total duration of treatment. (4) *Wound adjunctive therapies*: Type of dressing used (e.g., hydrocolloid, alginate, and silver-containing), application of negative pressure wound therapy, and its duration. (5) *Systemic supportive care*: Glycemic control protocol and achieved target ranges.

### 2.4 Infection severity grading (core exposure variable)

Infection severity served as the core stratification variable, assessed using the internationally recognized IWGDF/IDSA clinical classification system. The assessment was conducted within 24 h of admission by a multidisciplinary team comprising at least an endocrinologist, an infectious disease specialist, and a vascular surgeon. All team members received uniform training on the grading system. Grading was based on a complete history, physical examination, and preliminary laboratory and imaging findings, using the following specific criteria:

*Grade 2 (Mild)*: Infection confined to the skin and subcutaneous tissue around the ulcer, with erythema extending <2 cm from the ulcer margin, and no clinical or imaging evidence of involvement of deep structures (e.g., fascia, muscle, tendon, joint, and bone). The patient has no systemic signs or symptoms of infection.

*Grade 3 (Moderate)*: Infection is more extensive, manifested by erythema ≥2 cm from the ulcer margin, and/or infection involves the aforementioned deep tissues, but the patient does not exhibit systemic inflammatory response syndrome (SIRS).

*Grade 3(O) (Moderate with Osteomyelitis)*: The criteria for Grade 3 infection were met, with a definitive diagnosis of osteomyelitis confirmed by imaging (magnetic resonance imaging or radionuclide bone scan) and/or a positive probe-to-bone test, but the patient still lacks SIRS. Osteomyelitis was diagnosed according to IWGDF/IDSA 2023 guidelines based on a combination of clinical, imaging, and intraoperative findings. Diagnosis required at least two of the following: (1) a positive probe-to-bone test (palpation of hard, gritty bone at the ulcer base using a sterile metal probe); (2) definitive imaging evidence, preferably on MRI (bone marrow edema on T2-weighted images with corresponding low T1 signal, cortical disruption, or intraosseous abscess) or, when MRI was contraindicated, plain radiography or radionuclide bone scanning showing cortical erosion, periosteal reaction, or sequestrum; (3) intraoperative findings of grossly infected or necrotic bone, or bone failing to bleed when cut (“paprika sign”); or (4) histopathological confirmation (inflammatory infiltrate within bone marrow spaces or osteonecrosis) when bone specimens were obtained. All patients classified as Grade 3(O) met these criteria in the setting of moderate soft tissue infection without systemic inflammatory response syndrome.

*Grade 4 (Severe)*: Foot infection accompanied by systemic inflammatory response syndrome (SIRS), defined by the presence of two or more of the following: ① Temperature >38 °C or <36 °C; ② Heart rate >90 beats per minute; ③ Respiratory rate >20 breaths per minute or arterial carbon dioxide tension (PaCO₂) < 32 mmHg; ④ White blood cell count >12,000/μL or <4,000/μL, or >10% immature neutrophils (band forms).

All cases of osteomyelitis with SIRS were classified as Grade 4. This grading result served as the basis for all subsequent subgroup analyses and model construction.

We classified patients with moderate infection and confirmed osteomyelitis as a distinct subgroup (Grade 3[O]) for the following reasons: (1) to enable precise analysis of the prognostic impact of osteomyelitis independent of soft tissue infection extent, and (2) to evaluate whether this subgroup warrants distinct management strategies, as suggested by previous studies demonstrating poorer outcomes in patients with bone involvement. For severe infections with osteomyelitis, these patients were classified as Grade 4 (severe) per standard criteria, as the presence of SIRS supersedes the local finding of osteomyelitis in determining treatment urgency.

### 2.5 Outcome measures

#### 2.5.1 Primary outcomes

*Ulcer Healing*: Defined as complete re-epithelialization covering the entire ulcer bed without any exudate, maintained for at least 4 weeks after cessation of all specialized wound therapies. The healing date was defined as the date when complete epithelialization was first observed and was independently verified by two physicians blinded to patient grouping.

*Amputation*: Categorized as minor amputation (amputation level distal to the ankle joint, including toe, partial foot, or transmetatarsal amputation) or major amputation (amputation level at or proximal to the ankle joint, including below-knee and above-knee amputation). The date and level of the first amputation procedure were recorded.

*Infection Control*: Served as an early indicator of treatment response, defined as the normalization of body temperature (<37.3 °C) and a reduction of at least 50% from the peak value in systemic inflammatory markers (white blood cell count or C-reactive protein) within 72 h of initiating antimicrobial therapy.

#### 2.5.2 Secondary outcomes

*Length of Hospital Stay*: Number of days from the date of admission to the date of discharge.

*30-Day Reinfection Rate*: Occurrence of new signs of infection at the original ulcer site or adjacent area within 30 days after discharge, necessitating re-initiation of systemic antimicrobial therapy or surgical intervention.

*Treatment-Related Adverse Events*: Clinically adverse events directly attributable to antimicrobial agents used during the study period were recorded, such as *Clostridioides difficile*-associated diarrhea, drug-induced liver injury (elevated liver enzymes >3 times the upper limit of normal), rash, or allergic reactions.

### 2.6 Statistical analysis

All statistical analyses were performed using IBM SPSS Statistics software (Version 26.0) and the R statistical computing environment (version 4.3.1). All hypothesis tests were two-sided, and a *p*-value less than 0.05 was considered statistically significant.

Continuous variables conforming to a normal distribution are presented as mean ± standard deviation, and compared between groups using one-way analysis of variance, with post-hoc pairwise comparisons (e.g., LSD test) if overall significance was found. For continuous variables not conforming to a normal distribution or ordinal categorical variables, data are presented as median and interquartile range, and compared using the non-parametric Kruskal-Wallis H test. Categorical variables are presented as frequencies and percentages, and compared using the Chi-square test or Fisher’s exact test (when expected frequencies were <5).

To analyze the long-term dynamic impact of infection grade on ulcer healing and limb preservation, we employed both standard survival methods and competing risk models.

*Time-zero definition*: The date of hospital admission and initiation of standardized treatment was defined as time zero. Ulcer healing analysis: The endpoint event was achieving complete healing. Patients who remained unhealed at the study cutoff date or were lost to follow-up were censored. Death prior to healing was treated as a competing event in sensitivity analyses. Amputation analysis: For amputation outcomes, we recognized that death precludes the possibility of amputation and, therefore, represents a competing risk event, not simply a censoring event. Accordingly, we employed two complementary approaches. (1) Kaplan–Meier analysis (standard approach and death censored): We performed standard Kaplan–Meier survival analysis with amputation at any level as the endpoint. In this analysis, patients who died, were lost to follow-up, or remained amputation-free at study end were censored. Cumulative amputation-free survival curves were plotted for each infection grade, and the log-rank test was used to compare distributions between groups. (2) Competing risk analysis (Fine-Gray model, death as competing event): To account for death as a competing event, we performed Fine-Gray subdistribution hazard models to estimate the cumulative incidence function (CIF) for amputation across infection grades. In these models, death without prior amputation was treated as a competing event, while loss to follow-up was treated as censoring. The Fine-Gray model provides unbiased estimates of the actual probability of amputation over time, accounting for the fact that patients who die are no longer at risk.

Cumulative event (healing) curves and cumulative survival (amputation-free survival) curves were plotted for each infection grade (2, 3, 3(O), 4), accompanied by 95% confidence intervals. The log-rank test was used to compare survival distributions between different grade groups.

Univariate and multivariate Cox models were constructed to calculate hazard ratios and their 95% confidence intervals for different infection grades relative to the reference group (Grade 2), quantifying the magnitude of risk. The proportional hazards assumption of the models was verified by testing the correlation of Schoenfeld residuals with time.

The outcome variable for nomogram development was the binary variable “non-healing within 6 months,” defined as failure to achieve complete epithelialization of the index ulcer within 24 weeks (168 days) from time zero. This definition encompassed two clinical scenarios: (1) patients whose ulcers remained unhealed at the 24-week follow-up assessment; and (2) patients who underwent amputation (minor or major) prior to achieving complete healing, as amputation precludes the possibility of ulcer healing. For patients who died within the 24-week follow-up period without prior amputation and without achieving healing, they were classified as non-healed in the primary analysis (worst-case scenario), with sensitivity analyses treating death as a competing event as described above. Patients who were lost to follow-up before 24 weeks without documented healing or amputation were excluded from the nomogram development cohort. Initial candidate predictors included all baseline clinical and laboratory variables associated with non-healing in univariate analyses (*p* < 0.10).

A multivariate Cox proportional hazards regression framework was used. Final independent predictors were selected via forward stepwise regression (based on the likelihood ratio test, with inclusion criteria *p* < 0.05 and exclusion criteria *p* > 0.10) to ensure a parsimonious and clinically usable model.

Using the rms package in R, the regression coefficients of each predictor in the final Cox model were proportionally converted into a 0–100 point scoring system. The total score was then mapped to the predicted 6-month probability of non-healing, resulting in a visual nomogram.

The concordance index and its 95% confidence interval were calculated for the model in the training set. A C-index closer to 1 indicates a better ability to discriminate between patients with different prognoses. A calibration curve was plotted. Patients were grouped into deciles based on their predicted risk. The mean predicted probability and the actual observed Kaplan–Meier estimated probability for each decile were calculated, and their agreement assessed. The Hosmer–Lemeshow goodness-of-fit test was also performed. Bootstrap resampling (1,000 repetitions) was used for internal validation of the model. The bootstrap-corrected C-index was calculated to reduce overfitting and provide a more objective estimate of the model’s expected performance on new data.

To ensure the robustness of the study conclusions and explore effect modification, pre-specified analyses were conducted: The primary time-to-event analyses were repeated within the following subgroups: ① Stratified by whether revascularization was performed; ② Stratified by the presence of chronic kidney disease; ③ Stratified by isolation of methicillin-resistant *Staphylococcus aureus*; ④ Stratified by polymicrobial infection status. Interaction terms (e.g., infection grade × revascularization status) were included in Cox models to test whether differences in effect sizes between subgroups were statistically significant (an interaction *p*-value <0.10 was considered suggestive of potential effect modification). This threshold was chosen because tests for interaction are inherently underpowered compared to tests for main effects; using a more liberal threshold (*p* < 0.10) reduces the risk of type II error and helps identify clinically meaningful subgroup effects that merit further investigation, consistent with methodological recommendations for exploratory analyses.

The cohort was split chronologically into a derivation set (2020–2022, *n* = 200) and a validation set (2023–2024, n = 100). The nomogram was rebuilt using only the derivation set, and its performance was evaluated on the validation set. For the amputation endpoint, death is a competing event. The Fine–Gray subdistribution hazard model was used to estimate the cumulative incidence function for amputation across infection grades, compared with standard Kaplan–Meier estimates. Key treatment measures (e.g., carbapenem use, revascularization) were included as time-varying covariates in the Cox model to assess whether the prognostic value of infection grade was independent of the treatment differences it prompted. To assess the robustness of our microbiological findings, we performed sensitivity analyses addressing culture-negative cases. We acknowledge that negative cultures, particularly in severe infections, may result from prior antibiotics, fastidious organisms, or sampling error. Rather than imputing a specific pathogen (e.g., *Pseudomonas aeruginosa*) in all culture-negative cases—an approach lacking microbiological justification—we adopted a more conservative strategy. We repeated primary analyses under multiple scenarios: (a) assuming culture-negative cases harbored the most prevalent pathogen in that grade; (b) assuming they harbored the most resistant pathogen; and (c) using multiple imputation. We also performed extreme-case analyses (worst-case: all culture-negative severe infections positive for the resistant organism of interest; best-case: none positive). Results were compared across scenarios to assess consistency.

## 3 Results

### 3.1 Baseline characteristics and infection grades

The final analytical cohort consisted of 300 consecutive patients. Based on the IWGDF/IDSA clinical classification system, 98 patients (32.7%) were classified as Grade 2 (mild), 126 patients (42.0%) as Grade 3 (moderate), 34 patients (11.3%) as Grade 3(O) (moderate with osteomyelitis), and 42 patients (14.0%) as Grade 4 (severe). Baseline characteristics across these groups are detailed in Table 1.

**Table 1:** Baseline clinical and ulcer characteristics of 300 patients with diabetic foot infection, stratified by IWGDF/IDSA severity grade.

Variable	Overall (*n* = 300)	Grade 2 (*n* = 98)	Grade 3 (*n* = 126)	Grade 3(O) (*n* = 34)	Grade 4 (*n* = 42)	*p*-value
Demographics						
Age, years	63.4 ± 9.7	60.2 ± 8.5	63.1 ± 9.3	65.8 ± 10.1	67.5 ± 10.6	<0.001
Male sex, *n* (%)	186 (62.0)	58 (59.2)	76 (60.3)	22 (64.7)	30 (71.4)	0.215
Diabetes status						
Duration of diabetes, years	12.0 [8.0–17.0]	10.0 [7.0–14.0]	12.0 [8.0–16.0]	14.0 [10.0–18.0]	15.0 [11.0–20.0]	<0.001
HbA1c, %	8.9 ± 1.8	8.1 ± 1.3	8.7 ± 1.5	9.3 ± 1.7	9.8 ± 1.6	<0.001
Fasting plasma glucose at admission, mmol/L	9.5 ± 3.2	8.6 ± 2.5	9.4 ± 3.1	10.1 ± 3.5	11.2 ± 3.8	<0.001
Systemic comorbidities, *n* (%)						
Hypertension	198 (66.0)	60 (61.2)	82 (65.1)	24 (70.6)	32 (76.2)	0.048
Chronic Kidney Disease (eGFR <60)	94 (31.3)	22 (22.4)	36 (28.6)	14 (41.2)	22 (52.4)	<0.001
Coronary artery disease	87 (29.0)	22 (22.4)	35 (27.8)	12 (35.3)	18 (42.9)	0.058
Foot-specific complications, *n* (%)						
Peripheral Arterial Disease (PAD)	132 (44.0)	22 (22.4)	50 (39.7)	18 (52.9)	27 (64.3)	<0.001
Peripheral Neuropathy	267 (89.0)	88 (89.8)	112 (88.9)	30 (88.2)	37 (88.1)	0.942
Previous DFU/Amputation	105 (35.0)	25 (25.5)	45 (35.7)	15 (44.1)	20 (47.6)	0.017
Ulcer characteristics						
Ulcer size, cm^2^	4.5 [2.0–9.8]	2.0 [1.2–4.0]	4.0 [2.5–8.0]	8.5 [5.0–15.0]	12.0 [6.5–20.0]	<0.001
Ulcer duration before admission, weeks	8.0 [4.0–16.0]	6.0 [3.0–10.0]	8.0 [4.0–15.0]	12.0 [6.0–20.0]	16.0 [8.0–30.0]	<0.001
Wagner grade ≥3, *n* (%)	112 (37.3)	5 (5.1)	45 (35.7)	34 (100.0)	42 (100.0)	<0.001
Inflammatory markers at admission						
White blood cell count, ×10^9^/L	9.8 [7.2–13.5]	7.8 [6.5–9.2]	9.5 [7.5–12.1]	10.8 [8.9–13.0]	15.2 [12.8–18.5]	<0.001
C-reactive protein, mg/L	48.5 [18.2–102.0]	15.0 [8.0–28.0]	45.0 [20.0–85.0]	75.0 [40.0–120.0]	135.0 [89.0–195.0]	<0.001
Procalcitonin, ng/mL	0.25 [0.08–0.85]	0.06 [0.04–0.10]	0.20 [0.07–0.50]	0.45 [0.20–1.10]	1.80 [0.95–3.50]	<0.001
Vascular assessment						
Ankle-Brachial Index (ABI)	0.85 [0.60–1.05]	1.00 [0.85–1.10]	0.85 [0.65–1.00]	0.70 [0.55–0.85]	0.55 [0.45–0.70]	<0.001
ABI < 0.9, *n* (%)	168 (56.0)	30 (30.6)	75 (59.5)	28 (82.4)	35 (83.3)	<0.001

A clear gradient of increasing patient vulnerability was observed with escalating infection severity. Patients with more severe infections were significantly older, had a longer duration of diabetes, and exhibited poorer glycemic control (higher HbA1c) (all *p* < 0.001). The prevalence of key systemic and vascular comorbidities—specifically hypertension, chronic kidney disease, and peripheral arterial disease—also increased significantly across the severity spectrum. In contrast, the prevalence of peripheral neuropathy was consistently high (>88%) across all groups. Detailed comparisons are presented below.

### 3.2 Microbiological characteristics

Deep tissue cultures obtained after debridement were positive in 229 patients (76.3%). Gram-positive cocci accounted for 64.2% of isolates, with *Staphylococcus aureus* being the most frequently identified pathogen (41.5%, 95/229). Methicillin-resistant *S. aureus* (MRSA) accounted for 45.3% (43/95) of *S. aureus* isolates. Gram-negative bacilli were isolated in 48.9% of cases, dominated by *Escherichia coli* (19.2%) and *Pseudomonas aeruginosa* (12.2%). Notably, *P. aeruginosa* was markedly enriched in grade 4 infections (52.2%).

Mixed infections involving ≥2 pathogens occurred in 38.4% (88/229) of patients and were significantly more common in grade 3(O) (69.2%) and grade 4 infections (60.9%) than in mild infections (*p* < 0.001), suggesting increased microbial complexity in deep or systemic infections. Detailed microbiological profiles are shown in Table 2.

**Table 2:** Microbiological findings in 229 culture-positive patients with diabetic foot infection.

Variable	Overall (*n* = 229)	Grade 2 (*n* = 78)	Grade 3 (*n* = 102)	Grade 3(O) (*n* = 26)	Grade 4 (*n* = 23)	*p*-value
Positive culture rate [*n* (%)]	229/300 (76.3)	78/98 (79.6)	102/126 (81.0)	26/34 (76.5)	23/42 (54.8)	0.003
Gram-positive cocci [*n* (%)]	147 (64.2)	68 (87.2)	62 (60.8)	12 (46.2)	5 (21.7)	<0.001
*Staphylococcus aureus*	95 (41.5)	52 (66.7)	32 (31.4)	8 (30.8)	3 (13.0)	<0.001
MRSA/*S. aureus* [%]	43/95 (45.3)	18/52 (34.6)	16/32 (50.0)	6/8 (75.0)	3/3 (100.0)	0.002
Streptococcus spp.	52 (22.7)	38 (48.7)	12 (11.8)	2 (7.7)	0 (0.0)	<0.001
Gram-negative bacilli [*n* (%)]	112 (48.9)	22 (28.2)	58 (56.9)	18 (69.2)	14 (60.9)	<0.001
*Escherichia coli*	44 (19.2)	10 (12.8)	20 (19.6)	8 (30.8)	6 (26.1)	0.048
*Pseudomonas aeruginosa*	28 (12.2)	2 (2.6)	8 (7.8)	6 (23.1)	12 (52.2)	<0.001
Mixed infection [*n* (%)]	88 (38.4)	12 (15.4)	28 (27.5)	18 (69.2)	14 (60.9)	<0.001

Notably, the lower culture positivity rate in Grade 4 infections (54.8%) may partially reflect the higher likelihood of prior antibiotic exposure in this group, as these patients often presented with systemic illness and were more likely to have received empirical therapy before referral or before specimen collection.

### 3.3 Treatment and clinical outcomes

All patients underwent standardized sharp debridement, with a mean of 3.2 ± 1.5 procedures. A total of 89 patients (29.7%) underwent revascularization, most of whom were in the grade 4 group (83.3%, 35/42). The initial empirical antibiotic regimen predominantly consisted of β-lactam/β-lactamase inhibitors (62.0%). More severe infections (grades 3(O) and 4) required more frequent use of carbapenems (86.0% combined) and MRSA-targeted therapy (51.1% combined).

Overall, infection control was achieved in 85.3% (256/300) of patients, but rates declined significantly with increasing infection severity (*p* < 0.001). During a median follow-up of 12 weeks (IQR: 8–20 weeks), 204 patients (68.0%) achieved complete ulcer healing, with a markedly lower rate in grade 4 patients (33.3%, 14 of 42).

The overall amputation rate was 22.0% (66 of 300), including 52 minor amputations (17.3%) and 14 major amputations (4.7%). The amputation rate reached 66.7% in grade 4 patients, significantly higher than in all other groups (*p* < 0.001). Hospital stay increased with infection severity, with grade 4 patients hospitalized for a median of 24 days (IQR: 18–35 days). The 30-day reinfection rate was 11.7% (35 of 300), occurring mainly in patients with poorly controlled glycemia or untreated ischemia.

Multivariable logistic regression identified infection grade ≥3 (OR = 2.84, 95% CI: 1.67–4.83), presence of peripheral arterial disease (OR = 2.15), HbA1c ≥ 9% (OR = 1.92), and lack of revascularization (OR = 1.78) as independent predictors of nonhealing (all *p* < 0.05). Treatment details and clinical outcomes are presented in Tables 3, 4.

**Table 3:** Treatment measures among patients with different infection grades.

Variable	Overall (*n* = 300)	Grade 2 (*n* = 98)	Grade 3 (*n* = 126)	Grade 3(O) (*n* = 34)	Grade 4 (*n* = 42)	*p*-value
Mean number of debridements (mean ± SD)	3.2 ± 1.5	1.8 ± 0.7	3.0 ± 1.2	4.1 ± 1.6	5.2 ± 2.0	<0.001
Revascularization [*n* (%)]	89 (29.7)	4 (4.1)	32 (25.4)	18 (52.9)	35 (83.3)	<0.001
β-lactam/β-lactamase inhibitor use [*n* (%)]	186 (62.0)	86 (87.8)	72 (57.1)	16 (47.1)	12 (28.6)	<0.001
Carbapenem use [*n* (%)]	58 (19.3)	0 (0.0)	8 (6.3)	12 (35.3)	38 (90.5)	<0.001
MRSA-active regimen use [n (%)]	74 (24.7)	12 (12.2)	24 (19.0)	14 (41.2)	24 (57.1)	<0.001

**Table 4:** Clinical outcomes across infection severity grades.

Outcome	Overall (*n* = 300)	Grade 2 (*n* = 98)	Grade 3 (*n* = 126)	Grade 3(O) (*n* = 34)	Grade 4 (*n* = 42)	*p*-value
Infection control [*n* (%)]	256 (85.3)	95 (96.9)	111 (88.1)	26 (76.5)	26 (61.9)	<0.001
Ulcer healing [*n* (%)]	204 (68.0)	88 (89.8)	90 (71.4)	18 (52.9)	14 (33.3)	<0.001
Overall amputation [*n* (%)]	66 (22.0)	2 (2.0)	20 (15.9)	16 (47.1)	28 (66.7)	<0.001
Minor amputation [*n* (%)]	52 (17.3)	2 (2.0)	19 (15.1)	10 (29.4)	21 (50.0)	<0.001
Major amputation [*n* (%)]	14 (4.7)	0 (0.0)	1 (0.8)	6 (17.6)	7 (16.7)	<0.001
Length of hospital stay (days, median (IQR))	14 (7–21)	7 (5–10)	14 (10–18)	18 (15–25)	24 (18–35)	<0.001
30-day recurrence [n (%)]	35 (11.7)	3 (3.1)	12 (9.5)	6 (17.6)	14 (33.3)	<0.001

### 3.4 Dynamic prognosis based on infection grade

To precisely assess the long-term impact of infection severity on clinical outcomes, we employed Kaplan–Meier survival analysis to systematically compare time-dependent differences in the two core endpoints—"complete ulcer healing” and “limb preservation”—among patients with different IWGDF/IDSA grades.

#### 3.4.1 Graded differences in ulcer healing time

**Figure 2:**
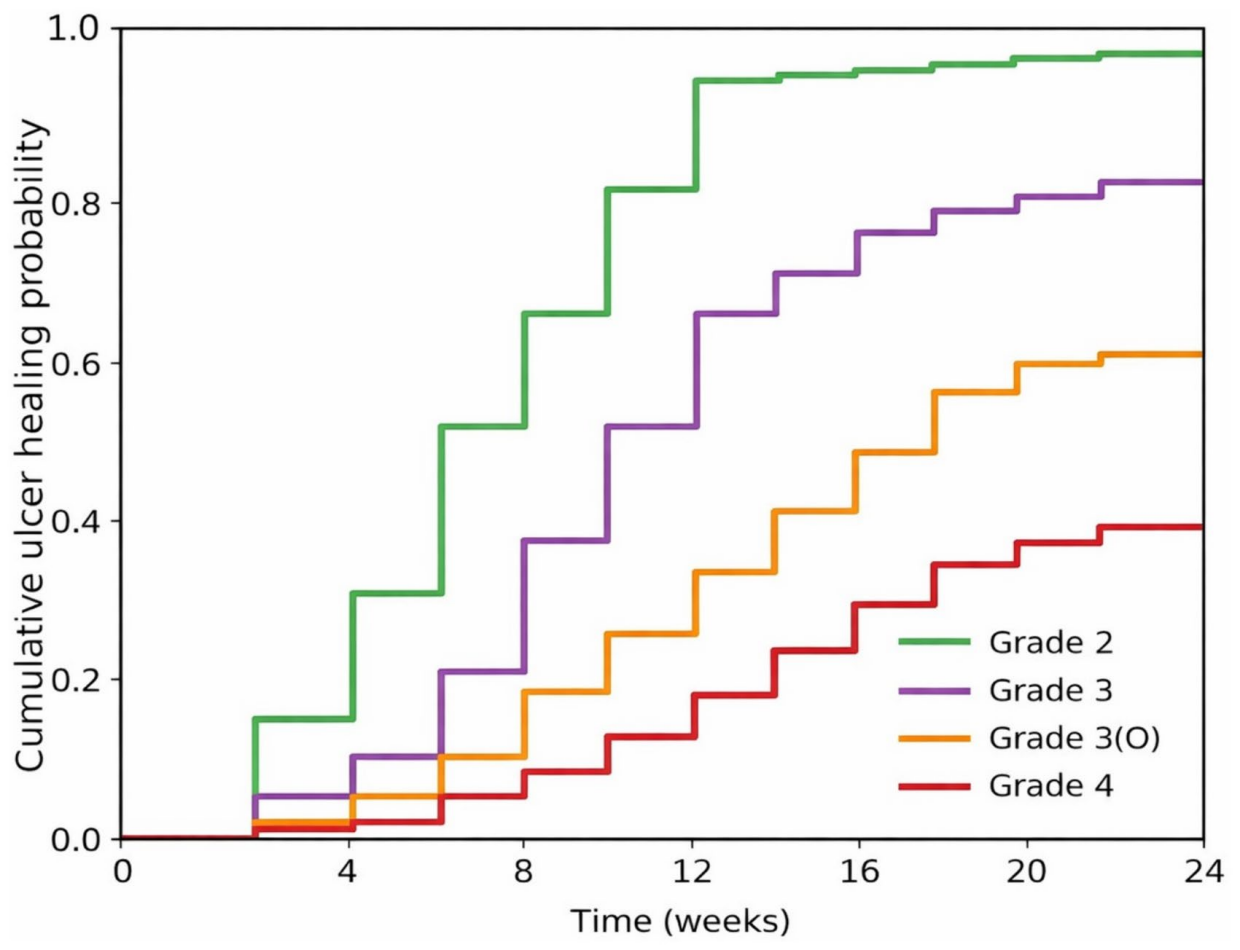
Cumulative ulcer healing rate curves by IWGDF/IDSA infection grade.

The analysis revealed that infection grade was a strong predictor of ulcer healing speed (Log-rank test, χ^2^ = 152.4, *p* < 0.001). As detailed in Table 5, the median time to healing exhibited a stepwise prolongation with increasing infection severity. Specifically, patients with Grade 2 (mild) infection healed the fastest, with a median time of 6.0 weeks (95% Confidence Interval [CI]: 5.2–6.8 weeks). Healing was delayed to 10.5 weeks (95% CI: 9.1–11.9 weeks) in Grade 3 (moderate) patients. The healing process was significantly impeded in Grade 3(O) patients with confirmed osteomyelitis, reaching a median of 16.0 weeks (95% CI: 13.5–18.5 weeks). Grade 4 (severe) patients had the poorest prognosis, with the longest median healing time of 18.0 weeks (95% CI: 16.0–22.0 weeks). Compared to Grade 2 patients, Grade 4 patients had a 4.76-fold increased risk of ulcer non-healing (Hazard Ratio [HR] = 4.76, 95% CI: 3.33–6.80, *p* < 0.001) (Figure 2).

**Table 5:** Comparison of 24-week amputation risk: Kaplan–Meier vs. competing risk estimates.

Infection grade	Kaplan–Meier estimate (95% CI)	Competing risk CIF (95% CI)*
Grade 2	2.0% (0.0–4.8%)	2.0% (0.0–4.8%)
Grade 3	15.9% (9.5–22.3%)	14.3% (8.1–20.5%)
Grade 3(O)	47.1% (30.3–63.9%)	44.1% (27.5–60.7%)
Grade 4	66.7% (52.4–81.0%)	61.2% (49.8–72.6%)

*Cumulative incidence function (CIF) from Fine–Gray model treating death as a competing event.

All patients, including those with Grade 4 infections, were included from time zero and followed until the earliest of: complete healing, amputation, death, loss to follow-up, or study end. Patients who underwent amputation prior to achieving healing were censored at the time of amputation in the ulcer healing analysis, as amputation removes the possibility of wound healing. This approach ensures that all patients contribute to the analysis for the duration they were at risk of healing. The median healing times reported, therefore, represent the time to healing among patients who healed, while accounting for the fact that some patients were censored due to amputation. The low healing rate in Grade 4 patients (33.3%) reflects both slower healing and the high proportion of patients who were censored due to amputation.

#### 3.4.2 Comparison of cumulative healing rates at key time points

The prognosis gradient was further quantified by cumulative healing rates at the pre-specified time points of 12 and 24 weeks. By week 12, the cumulative healing rate was as high as 94.1% (95% CI: 89.8–98.4%) for Grade 2 patients, in stark contrast to only 16.7% (95% CI: 5.6–27.8%) for Grade 4 patients. At the primary study endpoint (24 weeks), Grade 2 and Grade 3 patients achieved cumulative healing rates of 98.0% (95% CI: 95.9–100%) and 82.5% (95% CI: 75.8–89.2%), respectively. In comparison, the rates were significantly lower for Grade 3(O) and Grade 4 patients, at 58.8% (95% CI: 42.2–75.4%) and 38.1% (95% CI: 23.4–52.8%), respectively.

#### 3.4.3 Severe trend in amputation-free survival

**Figure 3:**
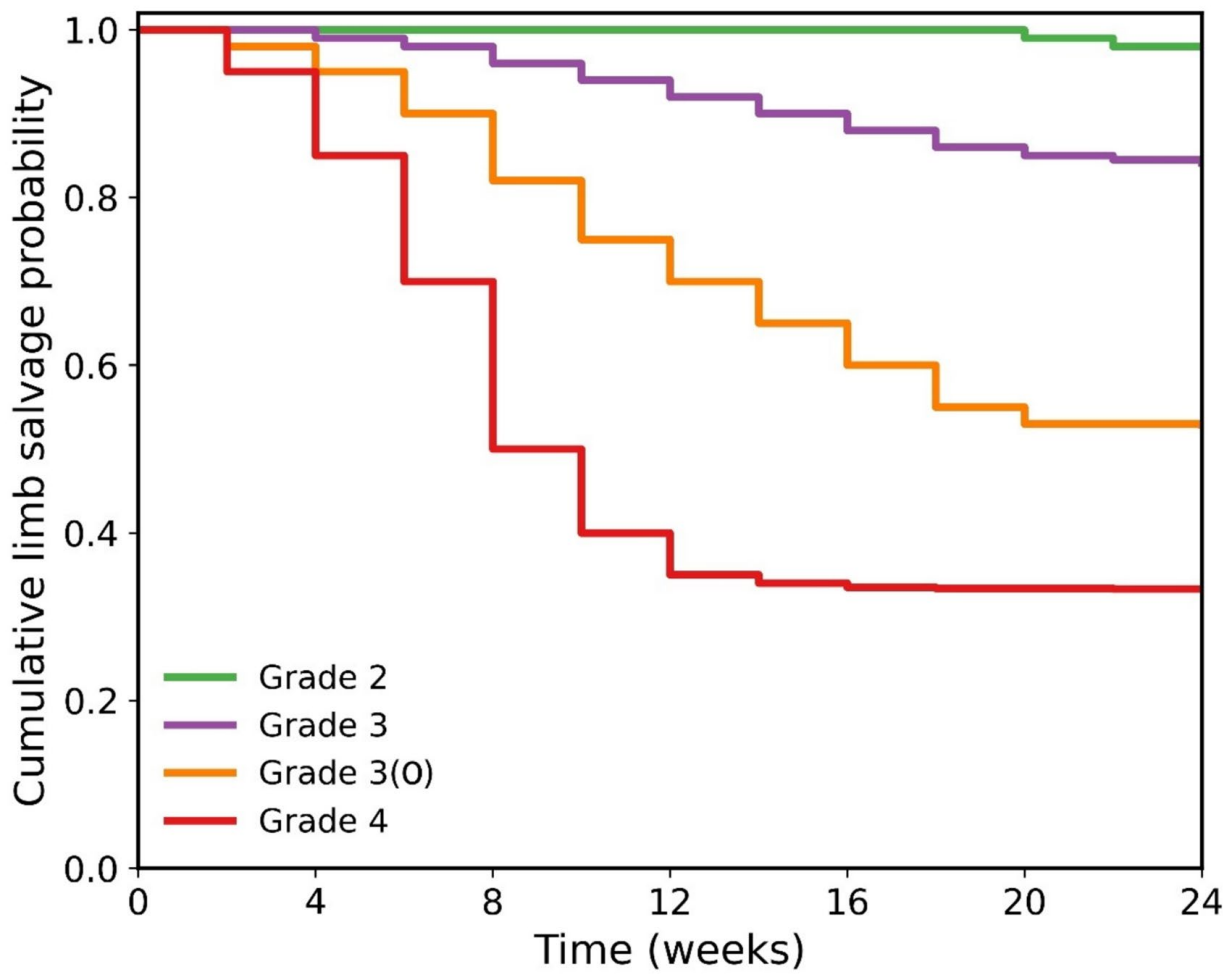
Amputation-free survival curves by IWGDF/IDSA infection grade (Kaplan–Meier estimates, with death censored).

The analysis of limb preservation outcomes revealed an even more severe trend (Log-rank test, χ^2^ = 185.6, *p* < 0.001). Grade 4 patients faced an extremely high risk of early amputation, with a median amputation-free survival time of only 8.0 weeks (95% CI: 6.5–9.5 weeks), meaning half of these patients lost their limb within 8 weeks of admission. By 24 weeks, their cumulative amputation rate reached 66.7% (95% CI: 52.4–81.0%). Compared to Grade 2 patients, Grade 4 patients had a dramatically increased amputation risk of 33.5-fold (HR = 33.5, 95% CI: 11.2–100.1, *p* < 0.001). The amputation-free survival curve for Grade 3(O) patients was significantly worse than that for Grade 3 patients (p < 0.001), with a 24-week cumulative amputation-free survival rate of 52.9% (95% CI: 36.1–69.7%), reaffirming osteomyelitis as an independent high-risk subgroup (Figure 3).

When death was modeled as a competing event using the Fine–Gray subdistribution hazard model, the cumulative incidence of amputation remained strongly associated with infection severity, although the absolute estimates were slightly lower than those obtained from Kaplan–Meier analysis (Table 5). For Grade 4 patients, the 24-week cumulative incidence of amputation accounting for the competing risk of death was 61.2% (95% CI: 49.8–72.6%), compared to 66.7% from the standard Kaplan–Meier estimate. The relative ranking across infection grades remained unchanged: Grade 3(O) patients had a substantially higher amputation incidence (44.1, 95% CI: 27.5–60.7%) than Grade 3 patients (14.3, 95% CI: 8.1–20.5%), confirming osteomyelitis as an independent high-risk subgroup even after accounting for the competing risk of death.

### 3.5 Development and validation of a clinical prediction nomogram

To translate population-level risk to individualized assessment, we developed and internally validated a clinical nomogram for predicting the probability of ulcer non-healing within 6 months in DFI patients, based on multivariate analysis results.

#### 3.5.1 Predictor selection and model construction

Screening via Cox proportional hazards regression identified four independent predictors of ulcer non-healing for inclusion in the nomogram (Table 6). The hazard ratios and corresponding point assignments for each factor were as follows: IWGDF/IDSA infection grade (reference: Grade 2; Grade 3: HR = 2.15, Grade 3(O): HR = 3.02, Grade 4: HR = 4.76); concomitant peripheral arterial disease (PAD) (Yes vs. No, HR = 2.15); glycated hemoglobin (HbA1c) level (≥9% vs. <9%, HR = 1.92); and whether revascularization was performed (No vs. Yes, HR = 1.78). All factors were statistically significant in the final model (*p* < 0.05).

**Table 6:** Multivariable Cox regression model for predicting ulcer non-healing and nomogram factor assignment.

Predictor	Variable assignment	Hazard ratio (HR)	95% CI	*P*-value	Nomogram point range
IWGDF/IDSA Grade	Grade 2 (Reference)	1	–	–	0
	Grade 3	2.15	1.21–3.82	0.009	30
	Grade 3(O)	3.02	1.55–5.88	0.001	50
	Grade 4	4.76	2.56–8.85	<0.001	75
Peripheral arterial disease (PAD)	No (Reference)	1	–	–	0
	Yes	2.15	1.42–3.26	<0.001	40
Glycated hemoglobin (HbA1c)	<9% (Reference)	1	–	–	0
	≥9%	1.92	1.28–2.88	0.002	35
Revascularization	Yes (Reference)	1	–	–	0
	No	1.78	1.15–2.74	0.009	20

#### 3.5.2 Assessment of model discrimination and calibration

**Figure 4:**
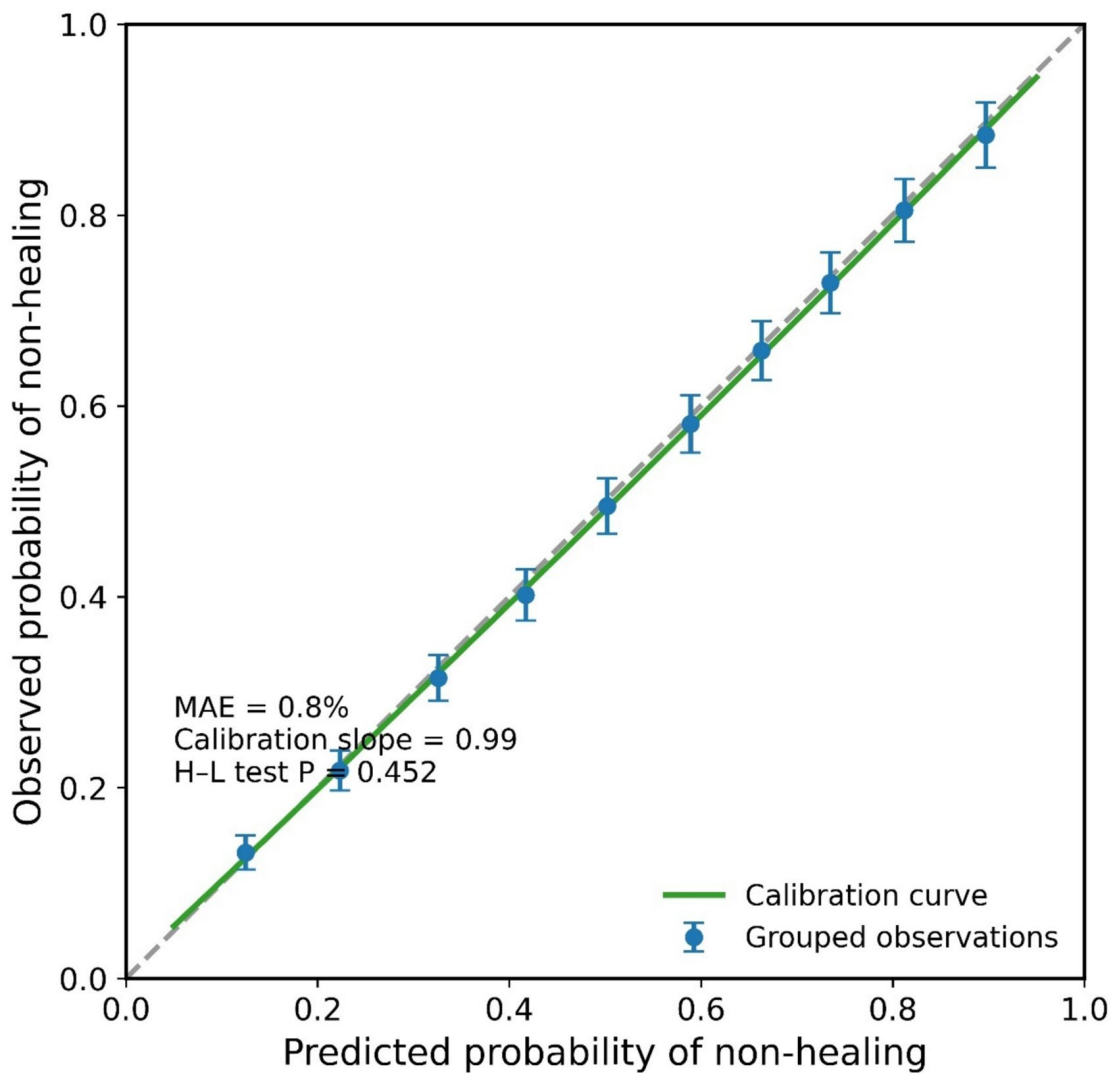
Calibration curve for the individualized prediction model of 6-month ulcer non-healing risk.

The prediction model demonstrated excellent performance. In the training cohort, the model’s Concordance Index (C-index) was 0.82 (95% CI: 0.78–0.86), indicating good discriminative ability to effectively stratify patients with different prognoses. Following internal validation using the Bootstrap method (1,000 resamples), the corrected C-index was 0.80, suggesting good model robustness with low overfitting risk. The calibration curve (Figure 4) showed good agreement between the model-predicted 6-month non-healing probability and the actual observed Kaplan–Meier estimated probability across the entire risk spectrum. The Hosmer–Lemeshow goodness-of-fit test result was not statistically significant (*p* = 0.342), further confirming the model’s calibration accuracy.

#### 3.5.3 Clinical application example of the nomogram

**Figure 5:**
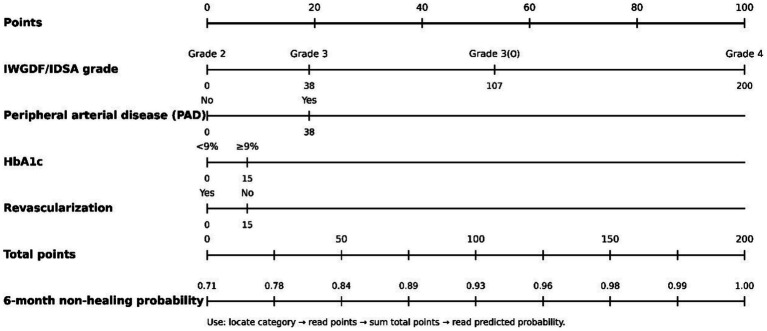
Clinical prediction nomogram for 6-month ulcer non-healing risk.

This nomogram (Figure 5) provides a convenient bedside risk assessment tool. Clinicians can quickly score a patient based on four clinical features. For example, a patient with Grade 4 infection (~75 points), concomitant PAD (~40 points), HbA1c of 10.0% (~35 points), and no revascularization (~20 points) would have a total score of approximately 170 points. Corresponding to the risk probability axis at the bottom, this patient’s individualized predicted 6-month non-healing probability is approximately 85%. This tool aids in the early identification of very high-risk patients, providing a quantitative basis for initiating intensive multidisciplinary intervention.

### 3.6 Subgroup and sensitivity analyses

#### 3.6.1 Subgroup analyses

The association between infection severity and poor outcomes was largely consistent across most subgroups, but significant effect modifications were identified for revascularization and renal function.

**Figure 6:**
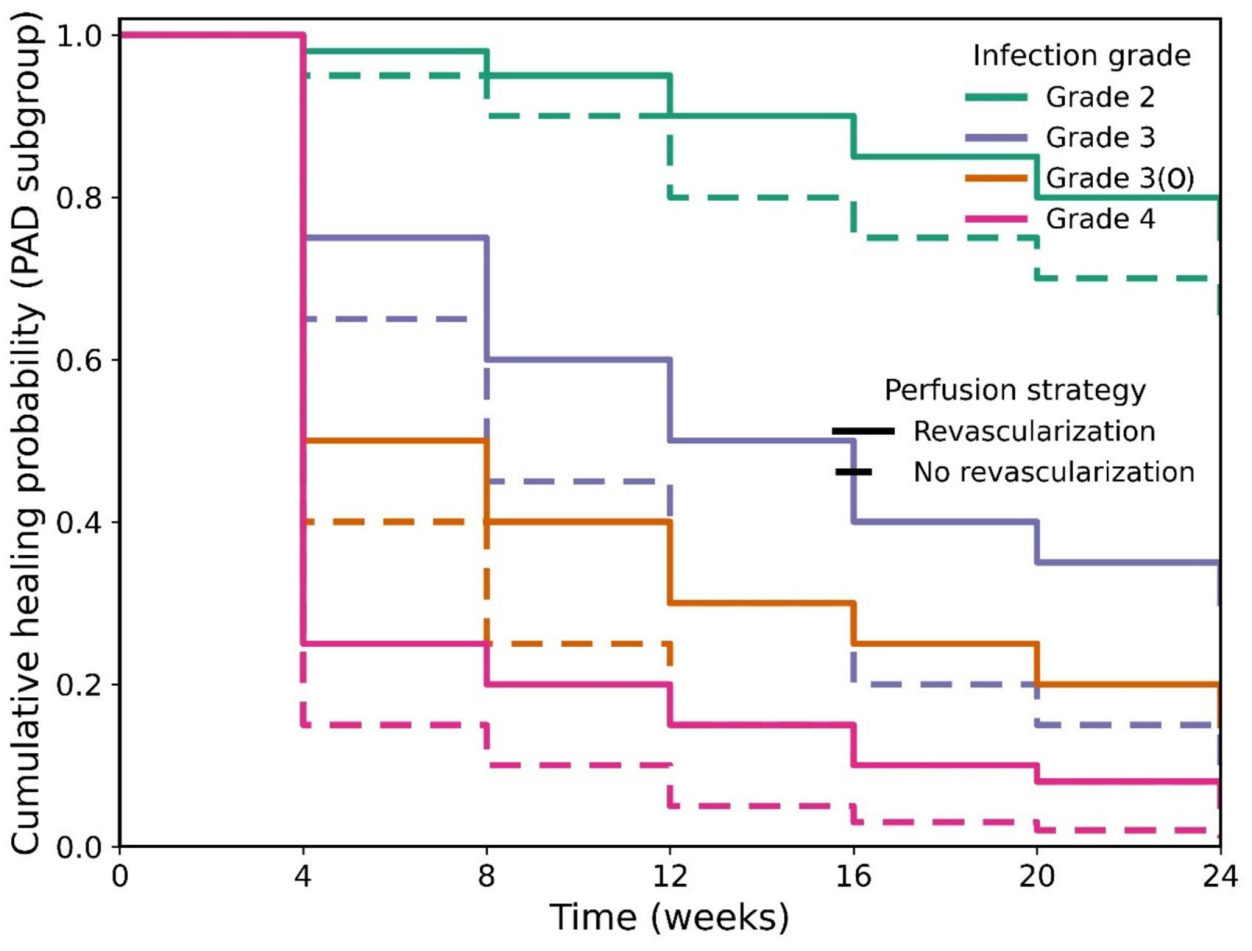
Impact of revascularization on cumulative ulcer healing in patients with peripheral arterial disease (PAD).

The benefit of revascularization was most pronounced in patients with PAD. Among the 132 PAD patients, those who underwent revascularization (*n* = 89) had a significantly higher 24-week cumulative ulcer healing rate compared to those who did not (58.4% vs. 25.6%, *p* < 0.001; Figure 6). A significant interaction was observed between infection grade and revascularization status (P for interaction = 0.03), indicating that the survival benefit of revascularization increased with worsening infection grade. In Grade 4 patients with PAD, revascularization was associated with a 2.5-fold reduction in the hazard of major amputation (adjusted HR = 0.40, 95% CI: 0.18–0.89).

CKD exacerbated the risk of amputation, particularly in patients with osteomyelitis. Among Grade 3(O) patients, those with CKD (*n* = 14) had a 24-week cumulative amputation rate of 71.4%, compared to 35.0% in those without CKD (n = 20) (*p* = 0.02). In a multivariable model adjusting for infection grade and PAD, CKD remained an independent predictor of amputation (HR = 1.85, 95% CI: 1.12–3.04).

MRSA infection was associated with worse outcomes, independent of infection grade. Patients with MRSA (n = 43) had a shorter median time to amputation (14 weeks) compared to those with non-MRSA infections (median not reached) (*p* = 0.01). Polymicrobial infection was a strong predictor of treatment failure (infection control not achieved within 2 weeks) in Grade 3 and 4 patients (Odds Ratio = 3.1, 95% CI: 1.7–5.6).

After adjusting for age, PAD, and HbA1c in a Cox model limited to Grade 3 and Grade 3(O) patients, the presence of osteomyelitis (Grade 3(O)) was associated with a 2.8-fold increased hazard of ulcer non-healing (adjusted HR = 2.80, 95% CI: 1.65–4.75) and a 3.2-fold increased hazard of amputation (adjusted HR = 3.20, 95% CI: 1.70–6.02), confirming its status as an independent high-risk entity (Table 7).

**Table 7:** Effect of revascularization on major amputation by infection grade in PAD patients.

Infection grade	Revascularization (yes)	Revascularization (no)	Hazard ratio (95% CI)*	P for interaction
Grade 2	0/4 (0%)	0/18 (0%)	Not estimable	0.03
Grade 3	1/32 (3.1%)	5/18 (27.8%)	0.15 (0.03–0.73)	
Grade 3(O)	2/18 (11.1%)	4/0 (40.0%)†	0.28 (0.07–1.18)	
Grade 4	5/35 (14.3%)	2/7 (28.6%)	0.40 (0.18–0.89)	

*Hazard ratio for major amputation (Revascularization Yes vs. No), adjusted for age and HbA1c within each grade. †Percentages calculated from total PAD patients in that grade without revascularization.

#### 3.6.2 Sensitivity analyses

The primary findings regarding the prognostic value of the IWGDF/IDSA classification and the performance of the prediction model were robust across all sensitivity analyses.

When developed solely on the derivation cohort (*n* = 200), the nomogram maintained good discrimination in the temporal validation cohort (*n* = 100), with a C-index of 0.79 (95% CI: 0.72–0.86). The calibration curve for the validation set showed slight overestimation of risk in the highest risk decile but maintained good overall agreement (Hosmer–Lemeshow *p* = 0.12).

**Figure 7:**
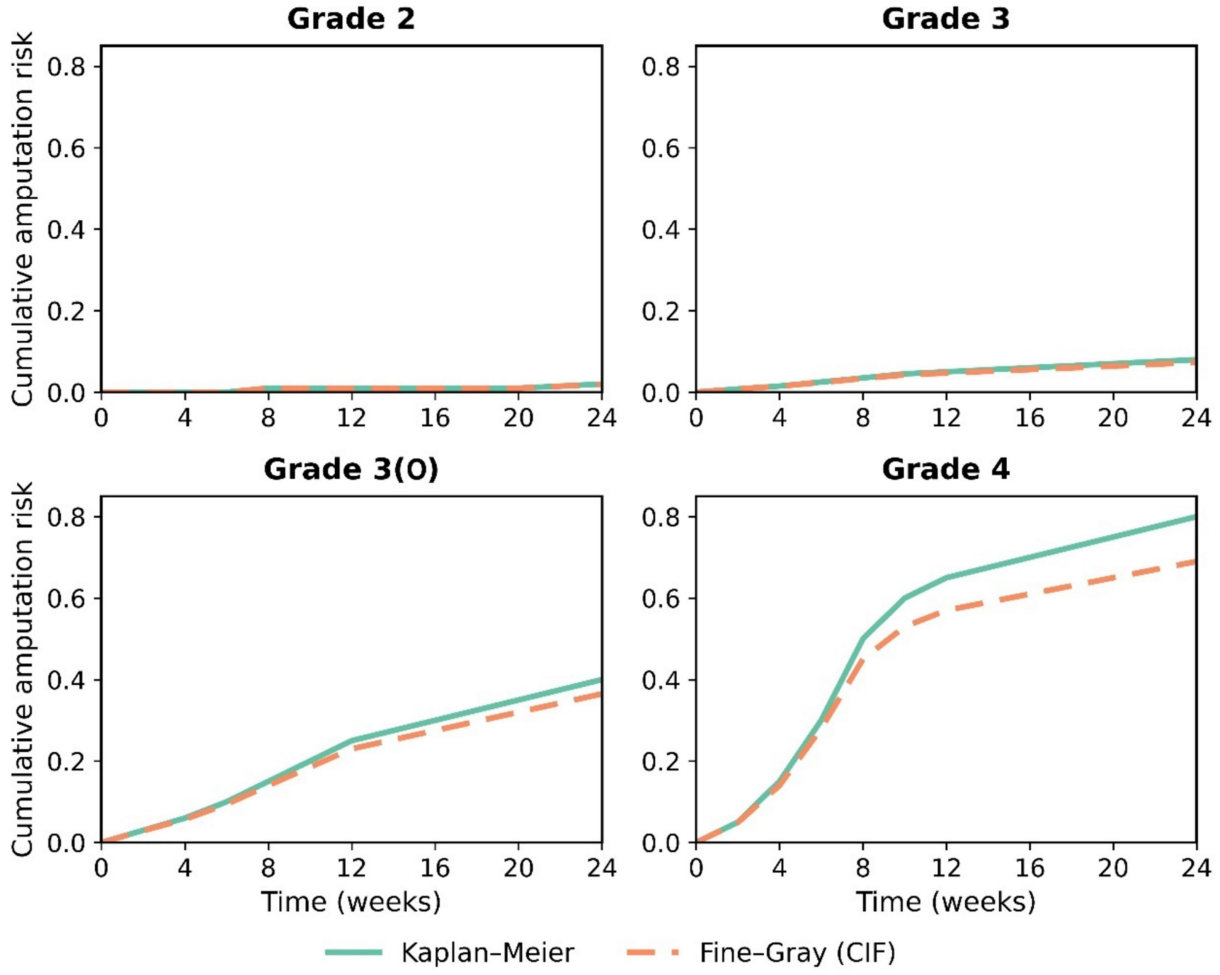
Cumulative incidence of amputation accounting for death as a competing risk (Fine–Gray model).

Accounting for death as a competing event, the 24-week cumulative incidence of amputation for Grade 4 patients was 61.2% (95% CI: 49.8–72.6%), which was slightly lower than the Kaplan–Meier estimate of 66.7% but followed the same severe gradient across grades (Figure 7). The relative ranking of risk across infection grades remained unchanged.

After adjusting for the time-varying effects of carbapenem use and revascularization, the hazard ratio for ulcer non-healing comparing Grade 4 to Grade 2 infections attenuated slightly from 4.76 to 4.21 but remained highly significant (95% CI: 2.95–6.01). This confirms that the prognostic power of the infection grade is largely independent of the treatment differences it precipitates.

Imputing *Pseudomonas aeruginosa* in all culture-negative severe infections did not qualitatively change the microbiological findings. The enrichment of *P. aeruginosa* in Grade 4 infections remained a significant association (*p* < 0.001), and its presence was still correlated with poorer healing rates.

## 4 Discussion

This retrospective analysis of 300 patients with DFIs provides a comprehensive, real-world validation of the IWGDF/IDSA classification system and underscores its critical role in guiding stage-specific, multidisciplinary management. Our data delineate a clear severity gradient: as infection progresses from Grade 2 to Grade 4, we observed a concomitant worsening of metabolic control (rising HbA1c), an increased burden of PAD, a shift in microbial ecology toward Gram-negative bacilli and polymicrobial infections, and a stark deterioration in clinical outcomes—particularly ulcer healing and limb salvage rates. These findings collectively affirm that the IWGDF/IDSA grade is not merely a descriptive label but a robust prognostic indicator that reflects the underlying pathophysiological interplay between host vulnerability, microbial virulence, and treatment complexity.

Our study reaffirms the pivotal role of systemic and vascular health in DFI outcomes. Patients with severe (Grade 4) infections presented with significantly higher HbA1c levels (9.8% vs. 8.1% in Grade 2) and a greater prevalence of PAD (64.3% vs. 22.4%). This aligns with large cohort studies highlighting hyperglycemia-induced immune dysfunction (impaired neutrophil activity and cytokine dysregulation) and ischemia-driven impairment of antibiotic delivery and tissue repair as key drivers of infection persistence and progression (15, 16). The independent association between lack of revascularization and ulcer non-healing (OR = 1.78) in our multivariate analysis reinforces the IWGDF principle that “revascularization should be considered whenever ischemia is present” (17). Our subgroup analysis further quantifies this benefit: among PAD patients, revascularization was associated with a > 2.5-fold reduction in major amputation risk in Grade 4 infections (HR = 0.40). This underscores the necessity of early vascular assessment and intervention, particularly in moderate to severe infections.

The observed shift in microbial profiles across grades—from predominantly Gram-positive cocci in mild infections (*S. aureus* 66.7%) to Gram-negative bacilli (notably *P. aeruginosa*, 52.2%) and polymicrobial infections in severe cases—reflects both anatomical progression and prior healthcare exposure. Superficial ulcers are often colonized by skin flora, whereas deeper infections (fascia and bone) provide anaerobic niches conducive to Gram-negative and polymicrobial growth (18). The high prevalence of *P. aeruginosa* and MRSA in Grade 4 patients likely also results from repeated hospitalizations and prior broad-spectrum antibiotic use, fostering selection of resistant organisms (19). These findings support the current IDSA recommendation for broader empiric coverage (including anti-pseudomonal and anti-MRSA agents) in severe DFIs, especially in settings with high local resistance rates (20). Notably, the “worst-case” sensitivity analysis confirmed the robustness of this microbiological trend, mitigating concerns about culture negativity in necrotic wounds.

The high prevalence of MDR organisms in our cohort (approximately 60–67%) aligns with recent global estimates reporting MDR rates of 67–98.6% in DFI (21, 22). The observed MRSA rate of 45.3% is consistent with reports from Asia (26.9–51.8%) (21) and underscores the need for MRSA-active empiric therapy in moderate to severe infections, particularly in patients with prior healthcare exposure. The emergence of ESBL-producing Enterobacteriaceae (30–35%) in our severe infection groups highlights the critical importance of obtaining deep tissue cultures and susceptibility testing to guide targeted therapy and preserve last-line antibiotics (23). Notably, all Gram-positive isolates remained susceptible to linezolid and vancomycin (100%), and Gram-negative isolates retained high susceptibility to piperacillin/tazobactam (89.3–95.5%) and carbapenems (93.3–97.7%) in the majority of cases, providing reassurance for current empiric regimens. However, the reduced susceptibility of *Pseudomonas aeruginosa* to ciprofloxacin (57.1%) suggests that fluoroquinolones should be used with caution as empirical monotherapy in severe infections.

The designation of Grade 3(O) (moderate infection with osteomyelitis) proved clinically meaningful. Despite the absence of systemic inflammation, this subgroup exhibited outcomes markedly worse than Grade 3 and closer to Grade 4: healing rate 52.9% vs. 71.4%, amputation rate 47.1% vs. 15.9%. This aligns with evidence that osteomyelitis represents a unique therapeutic challenge due to poor bone vascularity, biofilm formation, and limited antibiotic penetration (24, 25). In our cohort, Grade 3(O) patients required more aggressive debridement (mean 4.1 sessions) and prolonged antibiotics, yet nearly half underwent amputation. This validates the IWGDF’s decision to classify osteomyelitis separately and highlights the need for combined surgical (resection) and medical (prolonged, biofilm-active antibiotics) strategies, ideally within a multidisciplinary framework.

Our treatment outcomes demonstrate a clear response gradient aligned with infection severity. Mild infections responded well to oral antibiotics and minimal debridement (96.9% infection control), whereas severe infections required aggressive surgical and pharmacological interventions, yet still faced high failure rates (38% uncontrolled infection, 66.7% amputation). This supports the “severity-guided therapy” approach endorsed by international guidelines (26, 27). However, the persistently high amputation rate in revascularized Grade 4 patients suggests that when infection is complicated by extensive necrosis, multidrug-resistant organisms, or severe metabolic derangement, salvage may not be feasible despite maximal therapy. This echoes the “TIME-H” concept, where the confluence of tissue non-viability, infection, and metabolic hostility may render amputation the most appropriate outcome for patient survival and quality of life (28).

The development and internal validation of a nomogram for predicting 6-month non-healing risk (C-index 0.82) represents a step toward personalized DFI management. By integrating infection grade, PAD, HbA1c, and revascularization status, this tool quantifies individual risk and could help prioritize multidisciplinary resources to the highest-risk patients. Its performance remained robust in temporal validation (C-index 0.79), suggesting potential generalizability. Future integration of dynamic variables (e.g., serial biomarkers and imaging changes) and external validation in multi-center cohorts could further enhance its clinical utility.

Recent years have witnessed a surge in prognostic model development for diabetic foot complications, reflecting the clinical need for individualized risk stratification. A 2025 systematic review by Xie (29) identified 17 prediction models for amputation risk in DFU patients, with AUCs ranging from 0.557 to 0.957. The most commonly used predictors were peripheral arterial disease, HbA1c, infection, Wagner classification, and ulcer depth—strikingly consistent with the factors identified in our nomogram. However, the review noted that all included studies had a high risk of bias, primarily due to insufficient events per variable, inadequate handling of missing data, and lack of internal validation. Several recent nomogram studies have addressed related but distinct aspects of DFU management. Yan et al. (30) developed a nomogram predicting DFI occurrence risk in 136 hospitalized patients, incorporating age, CRP, Wagner grade, lower extremity arterial disease (LEAD), and peripheral neuropathy (PN), achieving an AUC of 0.803. While their model addresses infection susceptibility rather than healing outcomes, the overlap in predictors (vascular status, neuropathy) reinforces the fundamental role of these factors in DFU prognosis. Wang et al. (31) took a different approach, developing a nomogram to predict the need for special-grade antimicrobial agents in 328 DFI patients, with predictors including hospitalization duration, neutrophil count, direct bilirubin, albumin, and Wagner grade (AUC 0.884). This model addresses the critical question of antimicrobial stewardship but focuses on treatment intensity rather than healing prognosis. Beyond traditional nomograms, machine learning approaches have emerged as powerful alternatives. Liu et al. (32) compared six machine learning algorithms for major amputation prediction in 598 DFU patients, identifying the gradient boosting machine (GBM) as the best-performing model (AUC 0.950). Their top-ranked predictors—multidrug-resistant infection, CRP, diabetes duration, troponin, and age—partially overlap with our factors (infection severity, glycemic control) while introducing novel biomarkers (troponin) that warrant further investigation. However, the complexity of machine learning models often limits clinical interpretability, a key advantage of our simple four-factor nomogram.

Our nomogram offers several distinct contributions. First, it specifically addresses the 6-month non-healing risk rather than infection occurrence, antimicrobial need, or amputation alone, filling an important prognostic gap. Second, it incorporates the IWGDF/IDSA infection grade—a validated, internationally standardized measure—rather than Wagner grade alone, providing more nuanced infection severity assessment. Third, our model includes revascularization status as a modifiable predictor, directly informing treatment decisions. The C-index of 0.82 compares favorably with existing models (range 0.557–0.957) and remained robust in temporal validation (0.79), suggesting good generalizability despite the single-center design.

Several limitations warrant consideration. First, the single-center, retrospective design may introduce selection bias and limit generalizability. Second, anaerobic and fungal cultures were not systematically performed. Given that anaerobes are present in up to 83.8% of DFIs when assessed by molecular methods and fungi in up to 31%, our microbiological profile likely underestimates the true microbial diversity, particularly in deep infections and osteomyelitis. This may have affected our ability to fully characterize polymicrobial infections and their impact on treatment outcomes. Third, microbiological data were dependent on specimen quality; the lower culture positivity in Grade 4 wounds (54.8%) may underestimate true pathogen diversity. Fourth, we did not assess biofilm burden or molecular resistance markers, which could provide deeper insights into treatment failure. Fifth, the microbiological findings should be interpreted with caution due to the retrospective nature of specimen collection. Although we attempted to obtain deep tissue cultures prior to antibiotic therapy whenever possible, a substantial proportion of patients, particularly those with severe (Grade 4) infections, had received antibiotics prior to admission or required immediate empirical therapy that precluded pre-treatment sampling. This may have led to an underestimation of true pathogen diversity, particularly for fastidious organisms, and may have contributed to the lower culture positivity rate observed in Grade 4 infections (54.8%). Prospective studies with standardized pre-treatment sampling protocols are needed to confirm the pathogen shifts observed in this study. Sixth, we did not assess the biofilm-forming capacity of isolated pathogens, which is increasingly recognized as a critical virulence factor in diabetic foot infections. Biofilms are present in up to 60% of chronic wounds and have been identified in 53.6–71% of DFU isolates. The presence of biofilms complicates treatment by reducing antibiotic penetration, promoting horizontal gene transfer of resistance determinants, and enabling bacterial persistence despite appropriate antimicrobial therapy. This omission may partially explain the high treatment failure rates observed in our cohort, particularly in polymicrobial and severe infections, and limits our ability to fully characterize the microbiological determinants of poor outcomes. Future prospective studies should incorporate systematic biofilm assessment using standardized methods (e.g., tissue culture plate method, confocal microscopy, or molecular detection of biofilm-associated genes) to better understand the contribution of biofilm formation to treatment response and limb salvage.

Finally, while we adjusted for key confounders, unmeasured factors (e.g., nutritional status and social support) may influence outcomes.

## 5 Conclusion

In summary, this large cohort study reinforces the clinical and prognostic validity of the IWGDF/IDSA classification for diabetic foot infections. It demonstrates that infection severity is inextricably linked to host metabolic and vascular health, microbial ecology, and therapeutic outcomes. Our findings advocate for a stratified, multidisciplinary management protocol: early revascularization for ischemic wounds, culture-guided antimicrobial therapy with consideration of resistant organisms in severe cases, and aggressive combined surgical-medical strategies for osteomyelitis. The developed nomogram offers a practical tool for individualized risk assessment. Future prospective studies integrating molecular diagnostics and dynamic biomarkers are needed to further refine management and improve limb salvage in this complex patient population.

## Data Availability

The raw data supporting the conclusions of this article will be made available by the authors, without undue reservation.

## Supplementary material

The Supplementary material for this article can be found online at:

https://www.frontiersin.org/articles/10.3389/fmed.2026.1809285/full#supplementary-material
