# Supplementary material for: Staged management of infected diabetic foot ulcers: a 300-patient cohort study on prognostic grading, pathogen dynamics, and individualized risk prediction
Source: Front Med (Lausanne). 2026 Mar 27;13:1809285. doi: 10.3389/fmed.2026.1809285 (PMC13065669; doi:10.3389/fmed.2026.1809285)
Supplement: Supplementary file 1 [file Table_1.docx]

**Supplementary Table 1. Antimicrobial Susceptibility Profiles of Major Bacterial Isolates from Diabetic Foot Infections (n=229)**

| Organism (n) | Antibiotic | No. Tested | No. Sensitive | Sensitivity (%) |
| --- | --- | --- | --- | --- |
| Gram-positive bacteria |  |  |  |  |
| Staphylococcus aureus (n=95) | Penicillin | 95 | 8 | 8.40% |
|  | Cefoxitin (MRSA screening) | 95 | 52* | 54.7%* |
|  | Gentamicin | 95 | 78 | 82.10% |
|  | Ciprofloxacin | 95 | 65 | 68.40% |
|  | Trimethoprim/sulfamethoxazole | 95 | 81 | 85.30% |
|  | Clindamycin | 95 | 62 | 65.30% |
|  | Erythromycin | 95 | 45 | 47.40% |
|  | Rifampicin | 95 | 88 | 92.60% |
|  | Linezolid | 95 | 95 | 100% |
|  | Vancomycin | 95 | 95 | 100% |
|  | Teicoplanin | 95 | 95 | 100% |
| MRSA subset (n=43) | Penicillin | 43 | 0 | 0% |
|  | Gentamicin | 43 | 28 | 65.10% |
|  | Ciprofloxacin | 43 | 15 | 34.90% |
|  | Trimethoprim/sulfamethoxazole | 43 | 36 | 83.70% |
|  | Clindamycin | 43 | 12 | 27.90% |
|  | Erythromycin | 43 | 8 | 18.60% |
|  | Rifampicin | 43 | 38 | 88.40% |
|  | Linezolid | 43 | 43 | 100% |
|  | Vancomycin | 43 | 43 | 100% |
| Streptococcus spp. (n=52) | Penicillin | 52 | 50 | 96.20% |
|  | Ceftriaxone | 52 | 51 | 98.10% |
|  | Erythromycin | 52 | 28 | 53.80% |
|  | Clindamycin | 52 | 32 | 61.50% |
|  | Vancomycin | 52 | 52 | 100% |
| Enterococcus spp. (n=20) | Ampicillin | 20 | 14 | 70.00% |
|  | High-level Gentamicin | 20 | 12 | 60.00% |
|  | Vancomycin | 20 | 17 | 85.00% |
|  | Linezolid | 20 | 20 | 100% |
| Coagulase-negative staphylococci (n=25) | Cefoxitin | 25 | 8 | 32.00% |
|  | Vancomycin | 25 | 25 | 100% |
|  | Linezolid | 25 | 25 | 100% |
| Gram-negative bacteria |  |  |  |  |
| Escherichia coli (n=44) | Ampicillin | 44 | 9 | 20.50% |
|  | Amoxicillin/clavulanate | 44 | 26 | 59.10% |
|  | Piperacillin/tazobactam | 44 | 42 | 95.50% |
|  | Cefazolin | 44 | 20 | 45.50% |
|  | Ceftriaxone | 44 | 22 | 50.00% |
|  | Ceftazidime | 44 | 28 | 63.60% |
|  | Cefepime | 44 | 30 | 68.20% |
|  | Ertapenem | 44 | 42 | 95.50% |
|  | Imipenem | 44 | 43 | 97.70% |
|  | Meropenem | 44 | 43 | 97.70% |
|  | Gentamicin | 44 | 32 | 72.70% |
|  | Ciprofloxacin | 44 | 25 | 56.80% |
|  | Trimethoprim/sulfamethoxazole | 44 | 24 | 54.50% |
|  | Amikacin | 44 | 43 | 97.70% |
| Klebsiella pneumoniae (n=15) | Piperacillin/tazobactam | 15 | 14 | 93.30% |
|  | Ceftriaxone | 15 | 9 | 60.00% |
|  | Imipenem | 15 | 14 | 93.30% |
|  | Ciprofloxacin | 15 | 10 | 66.70% |
| Proteus mirabilis (n=10) | (Similar to Enterobacteriaceae panel) | 10 | - | - |
| Pseudomonas aeruginosa (n=28) | Piperacillin/tazobactam | 28 | 25 | 89.30% |
|  | Ceftazidime | 28 | 23 | 82.10% |
|  | Cefepime | 28 | 24 | 85.70% |
|  | Imipenem | 28 | 22 | 78.60% |
|  | Meropenem | 28 | 23 | 82.10% |
|  | Gentamicin | 28 | 20 | 71.40% |
|  | Tobramycin | 28 | 22 | 78.60% |
|  | Amikacin | 28 | 27 | 96.40% |
|  | Ciprofloxacin | 28 | 16 | 57.10% |
| Enterobacter spp. (n=12) | (Similar to Enterobacteriaceae panel) | 12 | - | - |
| Acinetobacter spp. (n=5) | Imipenem | 5 | 3 | 60.00% |
|  | Amikacin | 5 | 4 | 80.00% |
